# From Data Stewardship to Model Stewardship: Extending Governance Frameworks for AI Era Health Data Use

**DOI:** 10.2196/97859

**Published:** 2026-06-05

**Authors:** Leon Rozenblit, Steven Labkoff, Charles Safran

**Affiliations:** 1 Q.E.D. Institute, New Haven, CT, United States; 2 Division of Clinical Informatics, Beth Israel Deaconess Medical Center, 133 Brookline Avenue, HVMA Annex, Suite 2200, Boston, MA, 02215, United States, 1 617-278-8162; 3 Luminant Consulting, Stamford, CT, United States; 4 Department of Medicine, Harvard Medical School, Boston, MA, United States

**Keywords:** data stewardship, model stewardship, AI governance, electronic health records, decontextualization, secondary use, clinical tropism, health data ethics

## Abstract

Maris et al document important ethical challenges at the intersection of electronic health record data and artificial intelligence development, but existing governance frameworks designed for secondary data use are categorically insufficient for artificial intelligence model training, which creates persistent deployable artifacts that encode local clinical patterns as generalizable knowledge. Drawing on two decades of stewardship framework development, we propose extending governance from data stewardship to model stewardship.

## Introduction

Maris et al [1] make an empirical contribution to the ethics of health data use for artificial intelligence (AI), grounding four cross-cutting themes (privacy, public trust, fair representation, and responsible integration) in stakeholder perspectives from the LEAPfROG project. Their identification of “decontextualization” as a central challenge deserves particular attention. We write from the vantage of two decades of work on stewardship frameworks for health data. The American Medical Informatics Association (AMIA) national framework for secondary use [2], the National Committee on Vital and Health Statistics (NCVHS) stewardship report to the Department of Health and Human Services [3], and the elaboration of data stewardship principles [4] established core principles (accountability, chain of trust, transparency, data quality) that Maris et al’s [1] stakeholders independently rediscover. This convergence is validating but concerning: the principles hold, yet remain unoperationalized. We argue that AI model training represents a fundamentally new form of data use requiring a shift from data stewardship to model stewardship.

## AI Model Training Is Not Your Grandfather’s Secondary Use

Nearly 20 years ago, a national expert panel defined the secondary use of health data as uses beyond direct patient care, including research, quality measurement, public health surveillance, and commercial applications [2]. AI model training falls under this broad umbrella, but it differs from every use the framework’s architects envisioned. Traditional secondary uses analyze data and produce bounded findings; AI training creates persistent, deployable artifacts: models that may be commercialized globally, influence clinical decisions at scale, and embed the assumptions of their training context into every future prediction. A research study produces conclusions bounded by its methods and sample; a model trained on the same data produces an artifact with unbounded downstream reach and no expiration date.

The NCVHS [3] recommended abandoning “secondary use” as too imprecise for meaningful governance; advice even more apt today. AI training should be recognized as a qualitatively distinct category of secondary use, with stewardship requirements reflecting its unique characteristics: persistence, scalability, commercial deployment, and the encoding of institutional context as generalizable knowledge.

## The Challenge of Decontextualization

Maris et al [1] identify decontextualization as a cross-cutting ethical concern. We argue it is more fundamental than their analysis suggests: not one challenge among several, but the mechanism through which the others arise.

Electronic health record data encode not only clinical facts but institutional workflows, documentation practices, coding conventions, billing incentives, and resource constraints. Van der Lei’s [5] first law of medical informatics, that data should be used only for the purpose for which they were collected, takes on new force when the reuse creates persistent, deployable artifacts rather than bounded research findings.

**Figure 1.**
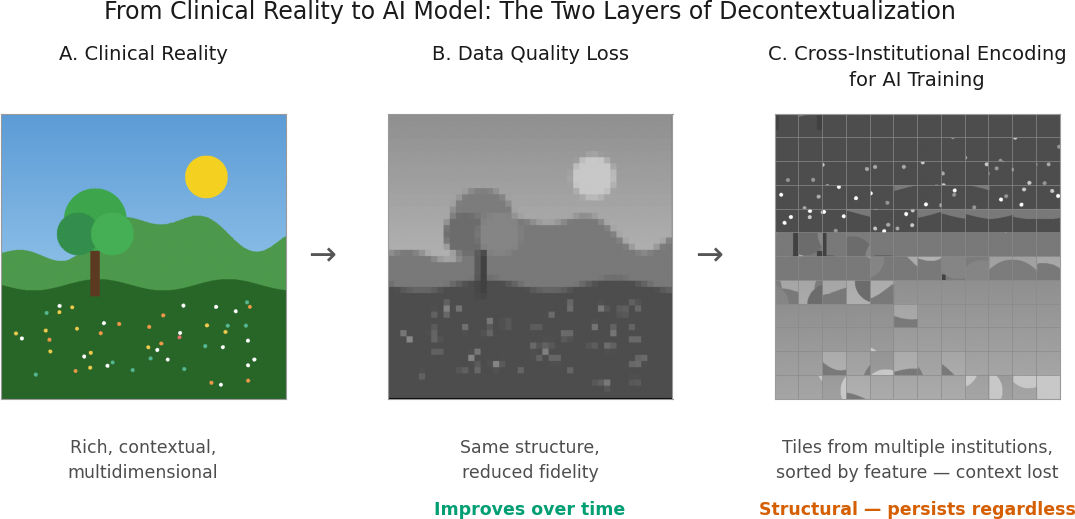
Progressive decontextualization of clinical data. Panel A: a recognizable scene representing the rich context of clinical reality. Panel B: the same scene reduced to a grayscale grid of discrete tiles. Panel C: the tiles resorted by value, severing all spatial relationships—a visual metaphor for how electronic health record data lose institutional context when extracted for model training. AI: artificial intelligence.

Two distinct challenges are at work. The first is data quality, and it is improvable: advances in ambient documentation, terminology that captures clinical intent, and better problem list governance will strengthen electronic health record data over time. The second is structural and persists regardless of data quality: every dataset carries the institutional fingerprint of its origin. What Maris et al [1], following Alami et al [6], term “clinical tropism,” the tendency of AI to reproduce narrow training environment practices, is a symptom of this structural layer. A model trained at an academic medical center with aggressive sepsis protocols learns different signals than one at a community hospital, not because the data are poor, but because they faithfully reflect different contexts (Figure 1).

Deploying models trained in one context across settings that differ systematically risks disadvantaging patients in predictable, preventable ways, the kind of harm that stewardship frameworks were designed to address.

## From Data Stewardship to Model Stewardship

The AMIA and NCVHS frameworks established stewardship principles for health data: accountability, chain of trust, transparency, oversight, data quality, and individual participation [3,4]. These principles must now extend to AI models and the datasets used to train them.

Consider the chain of trust, a core NCVHS concept. When data flow from hospital to aggregator to AI company to commercial model to clinical deployment across institutions, with the training data never represented, the chain does not merely stretch; it breaks. Who is the steward of a model trained on data from five health systems and deployed in 50?

Recent work offers concrete starting points that are achievable with existing infrastructure. Multistakeholder governance frameworks [7,8] propose domain-specific approaches: clinical decision support, real-world evidence generation, and consumer health AI each require distinct governance structures. The Safe, Effective, Equitable, Trustworthy (SEET) framework provides organizing principles [8], while recommendations for AI-enabled clinical decision support specify validation, certification, safety monitoring, adverse event reporting, and provenance documentation requirements [9,10]. Real-world data governance standards, including metadata requirements and bias documentation [9], offer complementary infrastructure.

Model stewardship, we propose, should encompass at minimum training data provenance documentation, so downstream users know what populations and practice settings a model reflects; (2) cross-institutional validation before deployment beyond the training context; (3) ongoing monitoring for context drift as clinical practices evolve; and (4) accountability structures that follow the model through its life cycle, not merely the data at its origin. All these requirements are technically feasible: provenance documentation and validation protocols exist in other regulated domains. Do we have the will to mandate them?

## Conclusion

Maris et al [1] are right that stakeholder-led governance is essential. But governance must evolve to match its target. AI models are not simply a new use of data; they are new artifacts with their own life cycle, risks, and accountability requirements. The stewardship frameworks built over two decades provide a proven foundation; extending them is the challenge. The immediate task is clear: require training data provenance and cross-institutional validation as preconditions for clinical AI deployment, just as we require evidence of efficacy before deploying therapeutics.

## Abbreviations

AI: artificial intelligence
AMIA: American Medical Informatics Association
NCVHS: National Committee on Vital and Health Statistics
SEET: Safe, Effective, Equitable, Trustworthy
